# CX3CL1-induced CD16^+^ monocytes extravasation in myeloperoxidase-ANCA-associated vasculitis correlates with renal damage

**DOI:** 10.3389/fimmu.2022.929244

**Published:** 2022-08-19

**Authors:** Jiale Tang, Zhonghua Liao, Liying Luo, Shuanglinzi Deng, Yuanyuan Jiang, Fangyuan Wang, Xinyue Hu, Hongling Yin, Guanghui Gong, Juntao Feng, Xiaozhao Li

**Affiliations:** ^1^ Department of Nephrology, Xiangya Hospital, Central South University, Changsha, China; ^2^ Center of Respiratory Medicine, Xiangya Hospital, Central South University, Changsha, China; ^3^ Department of Pathology, Xiangya Hospital, Central South University, Changsha, China

**Keywords:** antineutrophil cytoplasmic antibody (ANCA)-associated vasculitis, CD16^+^ monocyte, glomerular endothelial cells, CX3CL1, renal damage

## Abstract

**Background:**

Monocytes are involved in the pathogenesis of ANCA-associated vasculitis (AAV). Monocyte/macrophages are the dominant infiltrating cells in the glomeruli of patients with myeloperoxidase-AAV (MPO-AAV). However, how human monocyte subsets extravasate to the kidney in MPO-AAV with renal damage is unclear.

**Methods:**

30 MPO-AAV patients with renal damage and 22 healthy controls were enrolled in this study. Monocyte subsets and monocyte-related chemokines in the blood and kidneys of MPO-AAV patients were detected. The chemoattractant activity of the CX3CL1-CX3CR1 axis on CD16^+^ monocytes was observed. The effect of MPO-ANCA on the migration of CD16^+^ monocytes to human glomerular endothelial cells (HGECs) was detected by flow cytometry and transwell migration assay.

**Results:**

Compared with controls, CD16^+^ monocytes were significantly decreased in the blood and increased in the glomeruli of MPO-AAV patients with renal damage. The level of CX3CL1, but not CCL2, was significantly increased in the plasma of MPO-AAV patients. CX3CL1 co-localized with glomerular endothelial cells in MPO-AAV patients with renal damage. Moreover, we initially found that MPO-ANCA promotes an increase of the chemokine CX3CL1 on HGECs, imposing recruitment on CD16^+^ monocytes. Finally, the percentage of CD16^+^ monocytes in the blood was found to be positively correlated with estimated glomerular filtration rate (eGFR) and negatively correlated with urinary protein creatinine ratio in MPO-AAV patients with renal damage. Furthermore, the urinary protein creatinine ratio was positively correlated with the infiltrating of CD14^+^ and CD16^+^ cells in the kidneys.

**Conclusion:**

Enhanced extravasation of CD16^+^ monocytes to the kidney *via* the CX3CL1-CX3CR1 axis may be involved in renal damage in MPO-AAV.

## Introduction

The anti-neutrophil cytoplasmic antibody (ANCA)-associated vasculitis (AAV) is characterized by inflammation of blood vessels, endothelial injury and tissue damage (1). The AAV include microscopic polyangiitis (MPA), granulomatosis with polyangiitis (GPA) and eosinophilic granulomatosis with polyangiitis (EGPA). In addition, AAV is classified as myeloperoxidase (MPO)-ANCA vasculitis (MPO-AAV) and proteinase 3-ANCA vasculitis (PR3-AAV) based on autoantigen specificity (2).

Pathologically, AAV is characterized by inflammatory cells infiltrating the walls of small blood vessels, causing vascular damage and tissue necrosis. These inflammatory cells include neutrophils, T cells, macrophages, and monocytes (3). Although many studies have focused on neutrophils in the pathogenesis of AAV, monocytes also play an important role in AAV (4). Monocyte/macrophages are the dominant infiltrating cells in the glomeruli of patients with ANCA-associated glomerulonephritis (5). In a mouse model of anti-MPO antibody-induced necrotizing crescentic glomerulonephritis (NCGN), monocyte depletion significantly reduced glomerular necrosis and crescent formation (6). These studies suggest that monocytes are involved in the renal damage of AAV.

Human monocytes are divided into classical (CD14^++^CD16^-^), intermediate (CD14^+^CD16^+^), and non-classical (CD14^+^CD16^++^) subsets. Intermediate and non-classical monocytes are closely related in terms of gene expression profiles (7). Traditionally, human monocytes can be divided into CD16^-^monocytes (CD14^++^CD16^-^) and CD16^+^ monocytes (CD14^+^CD16^+^ and CD14^+^CD16^++^) (8). In a mouse model of crescentic glomerulonephritis, non-classical monocytes were observed to migrate to the glomeruli and interact with neutrophils to promote acute glomerular injury (9). However, which monocyte subsets migrate to the kidney involved in renal damage in MPO-AAV is unclear.

Monocytes migration from blood to tissue requires monocyte-endothelial interactions involving rolling, adhesion and extravasation. The expression of CD11b was increased in monocytes in AAV patients, and serum soluble markers of adhesion molecules were increased (10). These results suggest enhanced rolling and adhesion of monocytes to endothelial cells in AAV. However, the process of monocyte extravasation has not yet been studied.

Extravasation of monocytes into target organs is mediated by soluble chemokines, mainly CCL2 and CX3CL1 (8). CX3CL1 was mainly expressed in glomerular endothelial cells, which was associated with CD68^+^ macrophages but not with CD3^+^ T cell infiltration in AAV patients (11). Some CD68^+^ macrophages can be differentiated from peripheral blood monocytes (12). CX3CR1 is the only known corresponding CX3CL1 receptor (13). It suggests that the CX3CL1-CX3CR1 axis may be involved in monocytes extravasation to the kidney in MPO-AAV.

It is well known that ANCA plays an important role in the pathogenesis of AAV (14). Similar to neutrophils, monocytes also express MPO and PR3. Activated monocytes respond to ANCA by producing pro-inflammatory cytokines, chemokines, and reactive oxygen species (ROS) (15). *In vitro*, anti-MPO IgG induced CXCL-8 and CXCL-2 secretion by glomerular endothelial cells, leading to neutrophil chemotaxis (16). Whether MPO-ANCA can also affect the extravasation of monocyte subsets is unknown.

Here, we analyzed monocyte subset abundances and phenotypes in the blood and kidneys of MPO-AAV patients with renal damage, and explored the enhanced effect of the CX3CL1-CX3CR1 axis on glomerular endothelial cells recruiting CD16^+^ monocytes from circulation to the glomeruli in MPO-AAV.

## Materials and methods

### Subjects

Complete data for the 143 AAV patients (69 females, 74 males, average age: 60 ± 14 years) and 176 healthy controls (HC) (90 females, 80 males, average age: 53 ± 11 years) were collected at Xiangya Hospital from December 2012 to June 2020 (the clinical characteristics of AAV and HC are summarized in **Supplement Table S1**). For follow-up *in vitro* experiments, peripheral blood samples were obtained from 30 patients positive for MPO-ANCA, diagnosed with MPA according to ACR/EULAR 2017 Provisional Classification Criteria, and 22 HC between March 2019 and June 2020. All patients had renal damage in the presence of hematuria, proteinuria, and/or an elevation of serum creatinine. Eight of them underwent renal biopsy and renal tissues were collected. Clinical characteristics are shown in **Table 1**.

**Table 1:** Clinical and histopathological data of partial patients with blood drawing.

Characteristic	MPO-AAV Patients	HC	*p value*
N	30	22	
Age (years, mean ± SD)	63 ± 13	59 ± 15	*0.2413*
Gender (female/male)	14/16	12/10	
Monocytes (10^9^/L)	0.57 ± 0.18	0.39 ± 0.10	*<0.0001*
CD16^-^ monocytes (%)	81.81 ± 7.65	78.74 ± 7.66	*0.1671*
CD14^++^CD16^-^ monocytes (%)	81.81 ± 7.65	78.74 ± 7.66	*0.1671*
CD16^+^ monocytes (%)	8.89 ± 4.81	13.61 ± 6.80	*0.0060*
CD14^+^CD16^+^ monocytes (%)	5.60 ± 2.76	5.10 ± 3.79	*0.5903*
CD14^+^CD16^++^ monocytes (%)	3.28 ± 2.91	8.58 ± 5.71	*<0.0001*
BVAS [M ± Q]	18 [4]		
MPO-ANCA titer (U/mL) [M ± Q]	52.19 [60.62]		
CRP (mg/L)	15.70 [33.14]		
ESR (mm/h)	94.00 [58.00]		
Scr (μmol/L)	279.00 [416.15]		
eGFR (ml/min/1.73m^2^)	18.96 [19.85]		
Proteinuria (g/24h)	1.80 [3.95]		
Hematuria (n, %)	26 (87%)		
Urinary red blood cell (n/μL)	53.00 [181.25]		
Urinary protein/creatinine ratio (g/g)	2.83 [3.98]		
Extrarenal manifestations n (%)			
ENT	2 (7%)		
Pulmonary	26 (87%)		
Gastrointestinal	5 (17%)		
Nervous system	1 (3%)		

Data are expressed as the number, mean ± SD or M ± Q. M ± Q, median ± p75-p25; ANCA, myeloperoxidase- antineutrophil cytoplasmic antibody; MPO-AAV, MPO-ANCA vasculitis; HC, healthy control; BVAS, Birmingham Vasculitis Activity Score; CRP, C-reactive protein; ESR, erythrocyte sedimentation rate; Scr, serum creatinine; eGFR, estimated glomerular filtration rate; ENT, ear-nose-throat.

All MPO-AAV patients were not treated with glucocorticoids, immunosuppressants, or plasma exchange before samples collection. Patients were excluded if they had other immune-related diseases, malignancies, or infections. HC were obtained from the Physical Examination Center of Xiangya Hospital. They had no history of autoimmune disease, cancer, or any other inflammatory syndrome. Disease activity was scored according to the Birmingham vasculitis activity score (17, 18). The study was approved by the Ethics Committee of Xiangya Hospital, Central South University (2019030598).

### Flow cytometry

**Figure 1:**
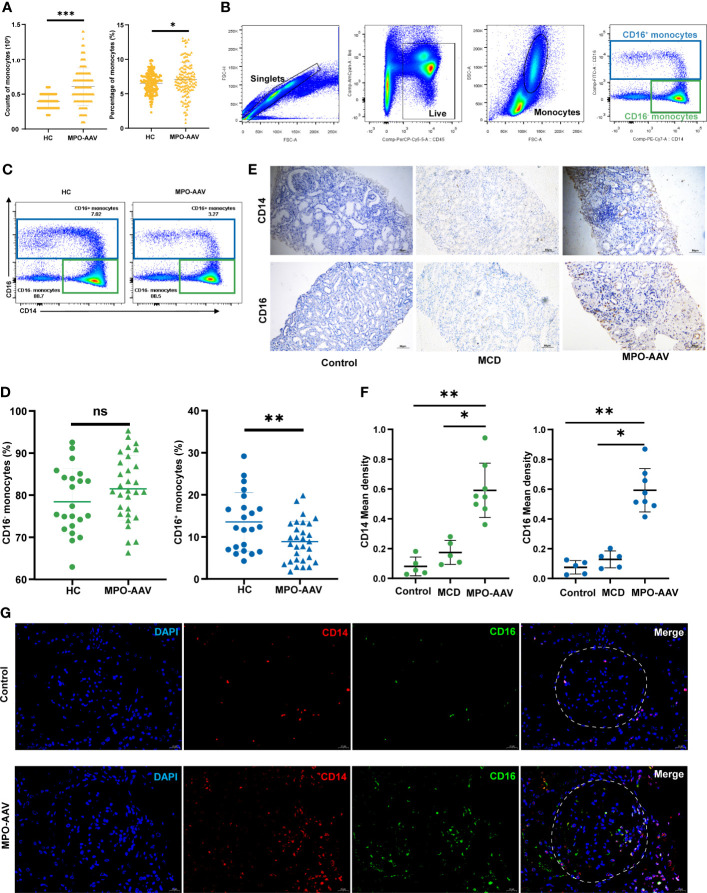
CD16^+^ monocytes abundance in the peripheral blood and kidneys of MPO-AAV patients with renal damage. **(A)** Significant differences in the monocytes in the blood of AAV patients and healthy controls (HC) were observed. **(B)** The abundance of monocyte subsets in PBMC from MPO-AAV patients (n=30) and HC (n=22) was analyzed by flow cytometry. Monocytes were identified within the FSC-A^hi^ and SSC-A^hi^ cell populations and the gate strategy was based on CD14 and CD16 expression; **(C)** Representative flow cytometry dot plots show the subsets of monocytes in MPO-AAV patients and HC; **(D)** The percentage of CD16^+^ and CD16^-^ monocytes in the peripheral blood of MPO-AAV patients and HC was shown. **(E, F)** Representative kidney sections showing CD14^+^ and CD16^+^ cells in MPO-AAV patients, patients with MCD, and control; **(F)** Quantification of CD14^+^ and CD16^+^ cell infiltrates in the kidneys of MPO-AAV patients (n=8), patients with MCD (n=5), and control (n=5). **(G)** CD14^+^CD16^+^ cells (orange) abundance was significantly increased in the glomeruli (white dotted line) of MPO-AAV patients than in control. ns, not significant. *p<0.05; **p<0.01; ***p<0.001.

Peripheral blood mononuclear cells (PBMCs) were isolated according to density gradient (Lymphoprep™ STEMCELL). Peripheral monocyte subsets were detected by flow cytometry. Surface staining was performed using a BD FACS Canto II flow cytometer. Data were analyzed with FlowJo software (version 10.0), and the gate strategy is described in **Figure 1B**. The following antibodies were used: Human TruStain FcX™, Zombie Aqua™ Fixable Viability Kit, anti-human CD45, CD16, CD14, CCR2, CX3CR1, CD40, CD80, and CD86 (all purchased from BD Biosciences or BioLegend). Negative thresholds for gating were set according to isotype-labeled controls.

### Immunocytochemistry

To fully characterize the infiltration of monocytes in the kidney, we performed immunohistochemical staining of paraffin-embedded kidney tissue specimens from MPO-AAV patients, patients with minimal change disease (MCD), and normal control. Normal control tissue was obtained from renal space-occupying lesions, and there was no evidence of tumor infiltration in pathology. Slides were deparaffinized by incubation at 60°C for 2-4 hours, followed by rehydration, where slides were immersed twice in xylol (15 minutes, each), transferred to 100% ethanol (5 minutes, each), once in 90% ethanol (5 minutes), 80% ethanol (5 minutes), 70% ethanol (5 minutes), and finally flushed three times with phosphate buffered saline (PBS) to wash away the ethanol. Next, antigen retrieval was performed with citrate (pH 6.0); nonspecific binding was blocked by incubation in 3% bovine serum albumin for 60 min at room temperature, and then the slides were incubated with mouse anti-human CD14 antibodies (ab182032, Abcam) or rabbit anti-human CD16 antibodies (ab203883, Abcam) for 18 h at 4°C. Antibody labeling was detected using an SP-HRP goat IgG kit (PV-6000, ZSGB-Bio, China) according to the manufacturer’s instructions. The chromogenic reaction solution contained 3,3’-diaminobenzidine (DAB) (ZLI-9018, ZSGB-Bio, China), and counterstaining was performed with Mayer’s hematoxylin (Solarbio, Beijing, China). The slides were viewed under fluorescent microscopy (Olympus BX51; Olympus, Tokyo, Japan).

The semi-quantitative of CD14^+^ and CD16^+^ cell infiltrates in the kidneys of controls and MPO-AAV patients was measured through the Image J program, and Mean density was used for statistical analysis.

### Immunofluorescence

Immunofluorescence staining was performed in kidney tissue embedded in paraffin according to standard pathology protocols. The primary antibodies used were mouse anti-human antibodies CD14 (ab182032, Abcam), CD31 (ab9498, Abcam), CCL2 (MABN712, Millipore), and rabbit anti-human antibodies CD16 (ab203883, Abcam), CD31 (ab32457, Abcam), CX3CL1 (ab85034, Abcam). The slides were placed in a wet chamber followed by the addition of the appropriate primary antibodies at the concentration recommended by the manufacturer (double staining) and incubated overnight at 4°C. The slides were rinsed three times with PBS, and a 1:200 dilution of Alexa Fluor^®^594 goat anti-mouse IgG antibody (ab150116, Abcam) and Alexa Fluor^®^488 goat anti-rabbit IgG antibody were applied and incubated at 37°C (30 minutes) in the dark. DAPI mounting medium (ab104139, Abcam) was used for nuclear staining. Finally, the slides were viewed under fluorescent microscopy (Olympus BX51; Olympus, Tokyo, Japan).

### Monocyte isolation

PBMCs from HC were extracted, and monocytes were isolated using magnetic CD14 microbeads (Miltenyi Biotech), according to the manufacturer’s instructions. CD16^+^ monocytes were isolated using CD16 microbeads (Miltenyi Biotech) after washing. The remaining cells were CD16^-^ monocytes.

### Purification of IgG

Blood or plasma exchange fluid was obtained from HC or MPO-AAV patients, and plasma was isolated and stored at –80°C. Control IgG and MPO-ANCA positive IgG were purified using protein G affinity chromatography. The preparation of IgG was performed according to previously described methods (19).

### Cells stimulation

Isolated monocytes were resuspended at 1 × 10^6^ cells/mL in RPMI 1640 medium (Gibco, Invitrogen, Carlsbad, CA) containing 10% heat-inactivated fetal bovine serum (FBS, Gibco). Monocytes were cultured in 6-well plates with or without 100 ng/mL LPS (Peprotech), MPO-ANCA, or control IgG at a final concentration of 250 μg/mL. The cells were incubated at 37°C for 24 h, after which cells were collected and stained with an antibody mix containing Human TruStain FcX™, Zombie Aqua™ Fixable Viability Kit, anti-human CD45, CD16, CD14, CCR2, and CX3CR1 (BioLegend), followed by flow cytometry. Data acquisition was performed using a BD FACS Canto II flow cytometer. Data were analyzed using FlowJo software (version 10.0).

Human glomerular endothelial cells (HGECs, ScienCell, San Diego, CA, USA) were cultured in endothelial cell medium (ECM, ScienCell) supplemented with 10% FBS, 1% penicillin/streptomycin, and 1% endothelial cell growth factor. HGECs were cultured in 12-well plates with or without 10ng/mL TNF-α (Peprotech), MPO-ANCA, or control IgG at a final concentration of 500 μg/mL. The cells were incubated at 37°C for 24h, after which total RNA of HGECs was extracted for polymerase chain reaction (PCR) amplification, and supernatants were collected and stored at -80°C for subsequent ELISA detection.

### Chemokine quantification

CCL2 and CX3CL1, measured by ELISA kits (eBioscience) according to the manufacturer’s protocols, were used to quantify monocyte chemokine levels in plasma and HGECs culture supernatant.

### Monocyte migration assay

Isolated monocytes (1 × 10^6^) were seeded into a transwell (Corning Costar Transwell^®^ 24 wells permeable 8 μM pore, Corning, NY, USA) to verify whether migration of monocyte subsets responded to different stimuli. Four different conditions were added to the lower chamber at 600 μl RPMI 1640 medium containing 10% FBS: chemokine free, 200 ng/mL CX3CL1 (C461, novoprotein), 200 ng/mL CCL2 (CM78, novoprotein) and 200ng/mL CX3CL1+2μg/mL anti-CX3CL1 monoclonal antibody (mAb) (AF365, R&D). Monocytes were incubated for 4 h at 37°C in a humidified atmosphere containing 5% CO2. Migratory cells were harvested and stained with an antibody mix containing Human TruStain FcX™, Zombie Aqua™ Fixable Viability Kit, anti-human CD45, CD16, and CD14, followed by flow cytometry. Data acquisition was performed using a BD FACS Canto II flow cytometer. Data were analyzed using FlowJo software (version 10.0). The migration index was calculated by dividing the number of monocytes that migrated in the chemokine-free group.

To further verify the migration effect of HGECs on CD16^+^ monocytes, CD16^+^ monocytes (6×10^5^) were added to the top chamber, supernatants from HGECs cultures with or without 1μg/mL anti-CX3CL1 mAb were placed in the bottom chamber at a volume of 600 μl, and the chambers were incubated at 37°C in a 5% CO2 atmosphere for 12 h. After incubation, the non-migratory cells in the upper chamber were scraped off, and the membrane was washed gently with PBS. The migratory cells on the bottom surface of the transwell membrane were fixed in 4% paraformaldehyde for 10 min, stained with Wright, and then viewed and photographed under a digital microscope (Olympus BX51; Olympus, Tokyo, Japan). The chemotaxis index was calculated by dividing the number of monocytes that migrated in response to the ECM.

### PCR

Total RNA was isolated using TRIzol reagent (Life Technologies, Ober-Olm, Germany) according to the manufacturer’s instructions. After quantification of the RNA concentration with Nanodrop (Thermo Scientific, Darmstadt, Germany), RNA samples were reverse transcribed at equal concentrations using a Takara First Strand cDNA Synthesis kit (Ambion, Foster City, CA) and then subjected to real-time qPCR analysis using Power SYBR Green (Applied Biosystems^®^ QuantStudio™ 7 Flex Real-time Fluorescent quantitative PCR system, Darmstadt, Germany). The comparative Ct method was used (2^–ΔΔCt^) for quantification. The primer sequences are listed in **Supplementary Table S2**.

### Statistical analysis

GraphPad Prism 8.0 software was used for statistical analysis. Data were expressed as the mean ± standard deviation. We performed the independent-sample t test or Mann-Whitney-U test to compare data between the two groups, the one-way analysis of variance (ANOVA) followed by Tukey multiple comparison test for multi-groups, and Spearman’s rank test for correlations. Experiments were repeated at least three times to ensure reproducibility, and differences were considered significant if *p* was less than 0.05.

## Results

### CD16^+^ monocytes were significantly decreased in the blood of MPO-AAV patients with renal damage

We analyzed the number and percentage of monocytes in the peripheral blood of AAV patients and HC. As shown, significant increases in the number and percentage of monocytes were observed in AAV patients compared to those in HC (**Figure 1A**). To further explore the altered total blood monocyte populations, we analyzed the circulating monocyte subsets of 30 MPO-AAV patients with renal damage by flow cytometry (**Figure 1B**). We found that the percentage of CD16^+^ monocytes in MPO-AAV patients was significantly decreased while the percentage of CD16^-^ monocytes (CD14^++^CD16^-^) was not significantly different compared to HC (**Figures 1C**, **D**). MPO-AAV patients exhibited a significantly decreased percentage of non-classical monocytes (CD14^+^CD16^++^) and no significant difference in the proportions of intermediate monocytes (CD14^+^CD16^+^) compared to HC (**Table 1**).

### Increased abundance of CD16^+^ monocytes in the kidneys of MPO-AAV patients with renal damage

To fully characterize the infiltration of monocyte subsets in the kidney, we performed immunohistochemistry and immunofluorescence staining of renal biopsy specimens from 8 MPO-AAV patients, 5 patients with MCD, and 5 normal control, respectively. Compared with MCD and normal control, the number of CD14^+^ and CD16^+^ cells in the kidneys of MPO-AAV patients was significantly increased (**Figures 1E**, **F**). The number of CD14^+^ CD16^+^ myeloid cells was significantly increased in the glomeruli and periglomerular of MPO-AAV patients compared to normal control (**Figure 1G**).

### The immunophenotype of monocyte subsets in MPO-AAV patients with renal damage

In order to investigate the reasons for changes in the distribution of monocyte subsets in MPO-AAV patients with renal damage, we explored the phenotypic differences of monocyte subsets in the peripheral blood.

**Figure 2:**
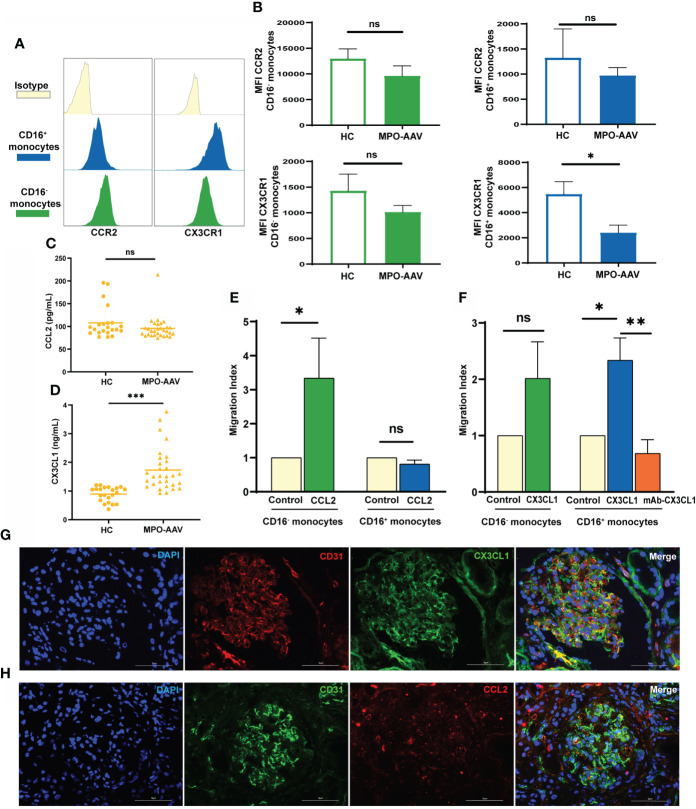
Altered CX3CL1-CX3CR1 axis and increased CD16^+^ monocytes migration in MPO-AAV patients with renal damage. **(A, B)** Histogram with plot diagrams of flow cytometry analysis show the mean fluorescence intensity (MFI) of CCR2 and CX3CR1 in monocyte subsets from MPO-AAV patients and HC. **(C)** The concentrations of CCL2 and **(D)** CX3CL1 were measured in the plasma of MPO-AAV patients with renal damage and HC. **(E)** *In vitro* migration assay of isolated monocytes from HC (n=6) with CCL2 and **(F)** CX3CL1, control wells were used as an indicator of conversion efficiency to calculate migration index. ns, not significant. *p<0.05; **p<0.01; ***p< 0.001. **(G)** Double immunofluorescence staining was performed to show glomerular endothelial cells (CD31, red) expressing CX3CL1 (green) and **(H)** glomerular endothelial cells (CD31, green) expressing CCL2 (red) in MPO-AAV patients. Nuclei were stained with DAPI (blue).

Consistent with most studies, out results demonstrated that CCR2 was mainly expressed in CD16^-^ monocytes, and CX3CR1 was mainly expressed in CD16^+^ monocytes (**Figure 2A**). Moreover, we observed decreased expression of CX3CR1 in CD16^+^ monocytes in MPO-AAV patients compared with HC. There was no significant difference in CCR2 expression in monocyte subsets from MPO-AAV patients compared with HC (**Figure 2B** and **Supplementary Figure S1**). Compared with CD16^-^ monocytes, CD16^+^ monocytes demonstrated higher expression of CD80 in MPO-AAV patients, and had an increased trend in the expression of CD86 and CD40. Overall, no differences were detected between the HC and MPO-AAV patients (**Supplementary Figure S2**).

### CX3CL1 was increased in the plasma of MPO-AAV patients with renal damage and enhanced CD16^+^ monocytes migration

We further detected the levels of CCL2 and CX3CL1 in the plasma of MPO-AAV patients with renal damage and HC. Compared with HC, CCL2 concentrations were not significantly different (**Figure 2C**), while CX3CL1 levels were significantly increased in the plasma of MPO-AAV patients (**Figure 2D**). Next, we evaluated the chemotaxis of CCL2 and CX3CL1 to monocyte subsets *in vitro* and receptor responsiveness to the ligand. We observed that CCL2 had no influence on CD16^+^ monocyte migration (**Figure 2E**). CX3CL1 did not influence CD16^-^ monocytes migration but increased CD16^+^ monocyte migration, and this effect was reversed by blocking CX3CL1 with a mAb (**Figure 2F**).

### MPO-ANCA can promote the recruitment of CD16^+^ monocytes by the CX3CL1-CX3CR1 axis in glomerular endothelial cells

To examine the expression of monocyte-related chemokines in the kidneys of MPO-AAV patients, we performed immunofluorescence staining for CX3CL1 and CCL2. We observed that CX3CL1 was significantly expressed in glomerular endothelial cells, while CCL2 was expressed in small amounts (**Figures 2G**, **H**).

**Figure 3:**
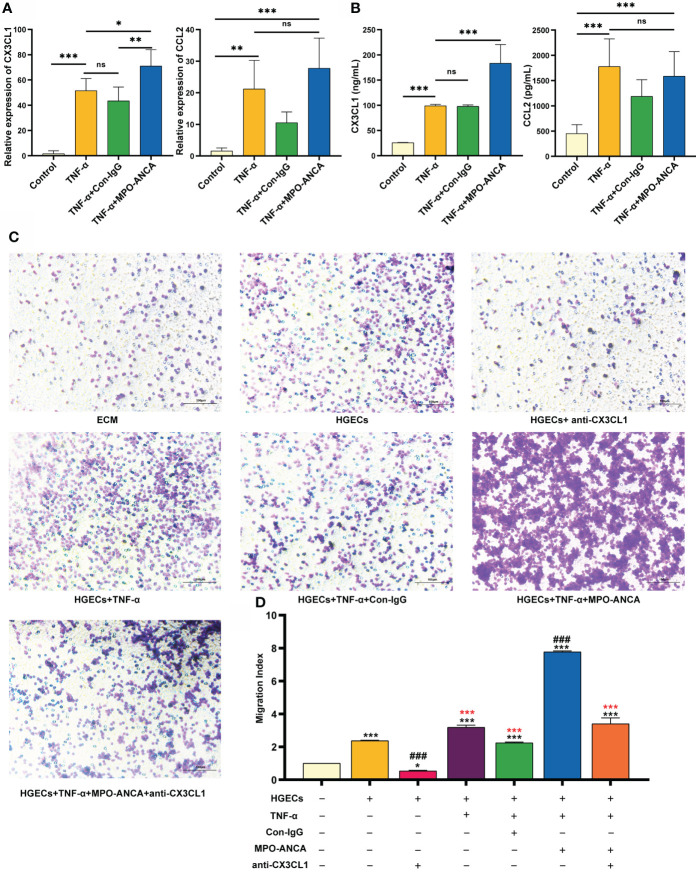
MPO-ANCA enhanced the recruitment of CD16^+^ monocytes by the CX3CL1-CX3CR1 axis in glomerular endothelial cells. **(A)** The production of CX3CL1 and CCL2 by HGECs stimulated with TNF-α, MPO-ANCA, or Con-IgG was measured by RT-qPCR and **(B)** ELISA. ns, not significant. *p<0.05; **p<0.01; ***p<0.001. **(C)** *In vitro* migration assay of CD16^+^ monocytes with the culture supernatants of HGECs, migrated CD16^+^ monocytes were stained by Wright staining. **(D)** ECM wells were used as an indicator of conversion efficiency to obtain migration index; *vs. ECM group, *p<0.05, ***p<0.001; ^#^vs. HGECs group, ^###^ *p*<0.001; *(red) vs. TNF-α+MPO-ANCA cultured HGECs group, ***(red)*p*<0.001. ECM, endothelial cell medium; HGECs, Human glomerular endothelial cells.

We further showed that the addition of MPO-ANCA led to a significant increase in CX3CL1 levels but no significant difference in CCL2 levels, in TNF-α-stimulated HGECs (**Figures 3A**, **B**). To explore the effect of MPO-ANCA on the migration of CD16^+^ monocytes to HGECs, the culture supernatant of the above groups of HGECs was used in a chemotaxis assay. The culture supernatant of unstimulated HGECs had a strong chemoattraction of circulating CD16^+^ monocytes, whereas anti-CX3CL1 mAb significantly inhibited monocyte chemotaxis. MPO-ANCA stimulation of HGECs increased CD16^+^ monocyte migration, and CX3CL1 neutralization almost completely prevented the migration (**Figures 3C**, **D**). No such effect was observed in the Con-IgG stimulation group (**Figure 3D**).

### CD16^+^ monocytes show more pronounced CX3CR1 upregulation in response to MPO-ANCA *in vitro*

**Figure 4:**
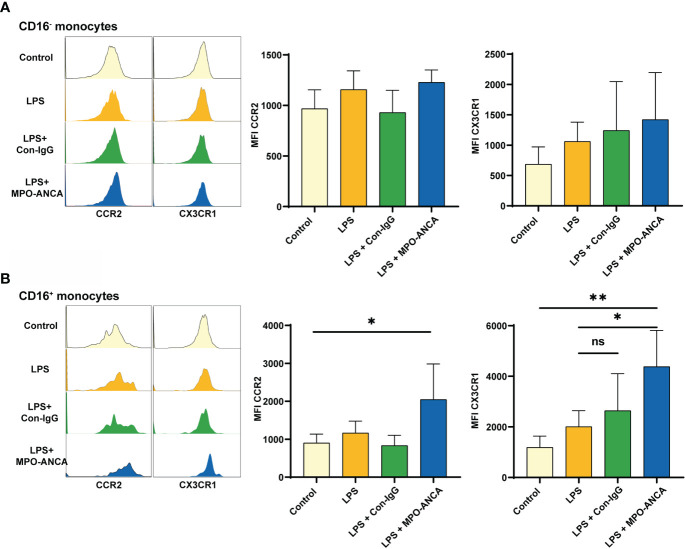
CD16^+^ monocytes show more pronounced CX3CR1 upregulation in response to MPO-ANCA *in vitro*. Isolated CD16^+^ and CD16^-^ monocytes were treated with LPS or combined with MPO-ANCA and Con-IgG for 24h, respectively. Experiments were performed with monocytes from 5 healthy donors; **(A)** the MFI of CCR2 and CX3CR1 in CD16^-^ monocytes after stimulation; **(B)** the MFI of CCR2 and CX3CR1 in CD16^+^ monocytes after stimulation. *p<0.05; **p<0.01. ns, not significant.

MPO in activated monocytes is transferred from the intracellular space to the surface and binds directly to ANCA. Since we observed differences in the expression of CCR2 and CX3CR1 in monocyte subsets between MPO-AAV patients and HC, we further explored the consequences of *in vitro* isolated CD16^+^ and CD16^-^ monocytes exposed to LPS and MPO-ANCA. We observed that CCR2 and CX3CR1 in CD16^-^ monocytes did not change significantly after stimulation (**Figure 4A**). However, the expression of chemokine receptors in CD16^+^ monocytes is more prone to change under stimuli. Both CCR2 and CX3CR1 in CD16^+^ monocytes were increased after LPS stimulation, and the increase in CCR2 and CX3CR1 was more significant after the combined addition of MPO-ANCA, while Con-IgG did not show this effect (**Figure 4B** and **Supplementary Figure S3**).

### Extravasation of CD16^+^ monocytes was associated with renal damage in MPO-AAV patients

**Figure 5:**
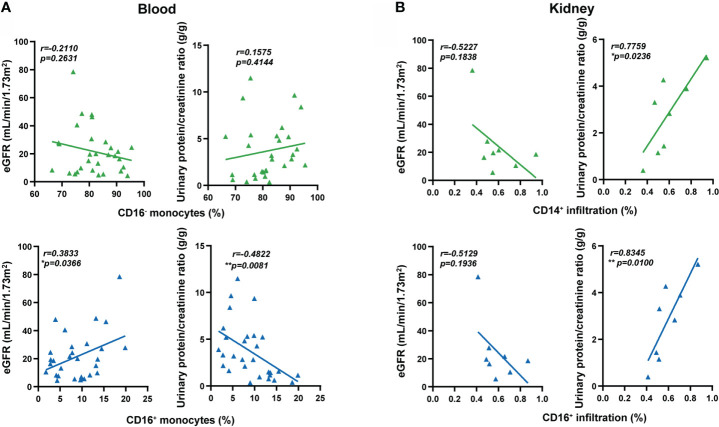
Extravasation of CD16^+^ monocytes was associated with renal damage in MPO-AAV patients. **(A)** The percentage of monocyte subsets (CD16^+^ and CD16^-^ monocytes) correlated with estimated glomerular filtration rate (eGFR, mL/min/1.73m^2^) and urinary protein/creatinine ratio (g/g) in MPO-AAV patients (n = 30); **(B)** The extent of CD14^+^ and CD16^+^ cell infiltrates in the kidneys of MPO-AAV patients (n = 8) correlated with eGFR and urinary protein/creatinine ratio, respectively. *p<0.05; **p<0.01.

Finally, we correlated the percentages of circulating monocytes with renal damage and disease activity. The low percentage of circulating CD16^+^ monocytes was correlated with low estimated glomerular filtration rate (eGFR) and high urinary protein creatinine ratio in MPO-AAV patients, but not with the percentage of CD16^-^ monocytes (**Figure 5A**). The percentage of monocyte subsets did not correlate with BVAS, ANCA titers, C-reactive protein (CRP), or erythrocyte sedimentation rate (ESR) (**Supplementary Figure S4**). To further reveal the possible role of CD16^+^ monocyte extravasation in renal damage, we analyzed the correlation between the extent of CD14^+^ and CD16^+^ cells infiltration in the kidneys and renal damage in MPO-AAV patients. The infiltration of CD14^+^ and CD16^+^ cells in the kidneys was positively correlated with urinary protein creatinine ratio in MPO-AAV patients with renal damage (**Figure 5B**). These data indicate that extravasation of CD16^+^ monocytes may exacerbate renal damage in MPO-AAV patients.

## Discussion

Even though it has been speculated that the CX3CL1-CX3CR1 axis may be involved in the evolution and progression of systemic vasculitis (20), the pathological role of the CX3CL1-CX3CR1 axis is poorly understood in AAV. Here, we demonstrate the functional implications of the CX3CL1-CX3CR1 axis on the abundance and recruitment of CD16^+^ monocytes in MPO-AAV with renal damage. We find an increased level of CX3CL1 in the plasma of MPO-AAV patients compared to HC. Accordingly, we observe a decrease in CD16^+^ monocytes in the peripheral blood and an increase in CD14^+^CD16^+^ cells infiltration in the kidneys of MPO-AAV patients compared with controls. Moreover, we document that MPO-ANCA promotes glomerular endothelial cell recruitment of CD16^+^ monocytes by enhancing the effect of the CX3CL1-CX3CR1 axis.

We found that compared with HC, the number and percentage of monocytes in peripheral blood of MPO-AAV patients with renal damage were increased, while the percentage of CD16^+^ monocytes was decreased. This discrepancy may be explained by the rapid extravasation of CD16^+^ monocytes into tissues during inflammation, resulting in circulating CD16^+^ monocytes consumption (13), and the release of classical monocytes from bone marrow to replenish circulating monocytes (21). In AAV patients, the percentage of monocyte subsets in the peripheral blood may vary in different clinical contexts (4). Previous studies on the percentage of circulating monocyte subsets were inconsistent with ours (22, 23), which may be related to the cause that all MPO-AAV patients collected in this study had renal damage and were not receiving corticosteroids or immunosuppressants. It has been reported that the percentage of CD14^++^CD16^+^ intermediate monocytes is enriched by glucocorticoid treatment (24, 25).

Compared with HC, the expression of CX3CR1 was increased in PBMCs, CD4^+^ T cells, and CD8^+^T cells, but there was no significant difference in the expression of CX3CR1 on monocytes in GPA patients (26). Similar results were also found in MPA patients (27). However, the above study did not analyze CX3CR1 expression in monocyte subsets. We observed that the expression of CX3CR1 on CD16^+^ monocytes was lower in MPO-AAV patients than in HC. In contrast, we found that MPO-ANCA can promote the surface expression of CX3CR1 on CD16^+^ monocytes *in vitro*. The expression of some chemokine receptors was decreased on the cell surface when they participate in chemotaxis (28). The CX3CR1 MFI was reduction on T cells after the addition of CX3CL1 *in vitro (* 29). This showed that in CX3CR1-expressing cells, ligand - receptor binding can result in decreased CX3CR1 surface expression. The significant decrease in CX3CR1 expression *in vivo* may be due to a loss of CX3CR1-expressing cells from the circulation or an interaction with CX3CL1. However, there are some flaws in our study. We did not explore CX3CR1 expression at the gene transcription level of CD16^+^ monocytes in AAV patients or the specific mechanism of upregulation of CX3CR1 on monocytes under MPO-ANCA stimulation *in vitro*. Therefore, further studies are required to understand the mechanism of CX3CR1 expression on monocytes in MPO-AAV.

We observed that the expression of CD80, CD86 and CD40 was increased on CD16^+^ monocytes compared with CD16^-^ monocytes, although to varying degrees. Importantly, previous studies showed that CD14^+^CD16^+^ monocytes produce the highest quantity of IL-1β in response to anti-MPO antibody stimulation (30). In this study, correspondingly, we found MPO-ANCA significantly upregulated CX3CR1 expression on CD16^+^ monocytes but had no effect on CD16^-^ monocytes, suggesting activated CD16^+^ monocytes react more strongly to MPO-ANCA than CD16^-^ monocytes. CD16^+^ monocytes are involved in many autoimmune diseases. The migration of circulating CD16^+^ monocytes to synovial tissue was increased in rheumatoid arthritis patients and was associated with joint destruction (31). Non-classical monocytes represent major immune intravascular cells contributing to glomerular inflammation and kidney injury in various mouse models and patients with lupus nephritis (32). Similar to previous findings, we observed that the infiltration of CD16^+^ monocytes in the kidneys was significantly increased compared with controls. Taken together, those results suggest that CD16^+^ monocytes have more proinflammatory effects in MPO-AAV patients with renal damage.

We found no difference in the levels of CCL2 and significantly increased levels of CX3CL1 in the plasma of MPO-AAV patients with renal damage compared to HC. Monocyte recruitment responds to chemokine receptor-ligand interactions (33). Our transwell migration assay demonstrated increased migration of CD16^+^ monocytes in the presence of CX3CL1 only, and this effect was reversed by anti-CX3CL1 mAb. Our results suggest that CX3CL1 rather than CCL2 drives increased specific migration of CD16^+^ monocytes.

Relative gene expression of CX3CL1 and CCL2 was upregulated in the kidney from the crescentic phase of anti-MPO IgG-treated mice compared with LPS-treated mice and was higher in glomeruli than in tubule-interstitial areas (34). High-CX3CL1 expression in the kidneys of AAV patients has been described (20). We further found that CX3CL1 was mainly expressed in glomerular endothelial cells in AAV patients. In addition, our *in vitro* results showed that MPO-ANCA from AAV patients contributed to the expression of mRNA and protein of CX3CL1 of glomerular endothelial cells induced by TNF-α. Subsequently, a migration assay further demonstrated that MPO-ANCA could enhance the recruitment of CD16^+^ monocytes by glomerular endothelial cells through the CX3CL1-CX3CR1 axis. Collectively, our results logically supplement the reported phenomenon that CX3CL1 expression is associated with monocyte/macrophages infiltration in the glomeruli of AAV patients (11).

The migration of CD16^+^ monocytes to tissues and the inflammatory responses they mediate, including the production of TNF-α and ROS, have the potential to aggravate autoimmune diseases such as lupus nephritis and rheumatoid arthritis (35). We found that decreased circulating CD16^+^ monocytes in MPO-AAV patients were positively correlated with eGFR and negatively correlated with urinary protein creatinine ratio. In line with this, we observed that the infiltration of CD14^+^ and CD16^+^ cells in the kidneys was positively correlated with proteinuria. Inhibition of CX3CR1 can reduce glomerular leukocyte infiltration and crescent formation in a rat model of crescent nephritis, thereby improving renal function (36).These suggest that CD16^+^ monocytes extravasate to the kidneys *via* the CX3CL1-CX3CR1 axis to participate in renal damage in MPO-AAV patients. However, to understand the role of CD16^+^ monocytes in renal inflammation in MPO-AAV, further studies are required.

In conclusion, this study identified that MPO-ANCA enhanced the role of CX3CL1-CX3CR1 axis, inducing extravasation of CD16^+^ monocytes to the kidneys to be involved in renal damage in MPO-AAV. Targeting the CX3CL1-CX3CR1 axis may aid in the management of MPO-AAV with renal damage.

## Data availability statement

The original contributions presented in the study are included in the article/**Supplementary Material**. Further inquiries can be directed to the corresponding author.

## Ethics statement

The studies involving human participants were reviewed and approved by Ethics Committee of Xiangya Hospital, Central South University (2019030598). The patients/participants provided their written informed consent to participate in this study.

## Author contributions

All authors contributed to the paper. JT, JF and XL designed research; JT, ZL, LL, SD, YJ, FW, XH and XL performed research; GG and HY contributed pathological analysis; XL supervised work; JT, ZL, XH, and SD analyzed data; and JT and XL wrote the paper. All authors contributed to the article and approved the submitted version.

## Funding

This work was supported by the Natural Science Foundation of Hunan Province (2020JJ4887), Clinical Medical Technology Innovation Guidance Program of Hunan Province (2020SK53701), and the Key Research and Development Program of Hunan Province (2021SK2033).

## Conflict of interest

The authors declare that the research was conducted in the absence of any commercial or financial relationships that could be construed as a potential conflict of interest.

## Publisher’s note

All claims expressed in this article are solely those of the authors and do not necessarily represent those of their affiliated organizations, or those of the publisher, the editors and the reviewers. Any product that may be evaluated in this article, or claim that may be made by its manufacturer, is not guaranteed or endorsed by the publisher.
