# Supplementary material for: CX3CL1-induced CD16+ monocytes extravasation in myeloperoxidase-ANCA-associated vasculitis correlates with renal damage
Source: Front Immunol. 2022 Aug 19;13:929244. doi: 10.3389/fimmu.2022.929244 (PMC9437287; doi:10.3389/fimmu.2022.929244)
Supplement: Supplementary file 1 [file Presentation_1.pdf]

Supplementary Material

1 Supplementary Figures and Tables

1.1 Supplementary Figures

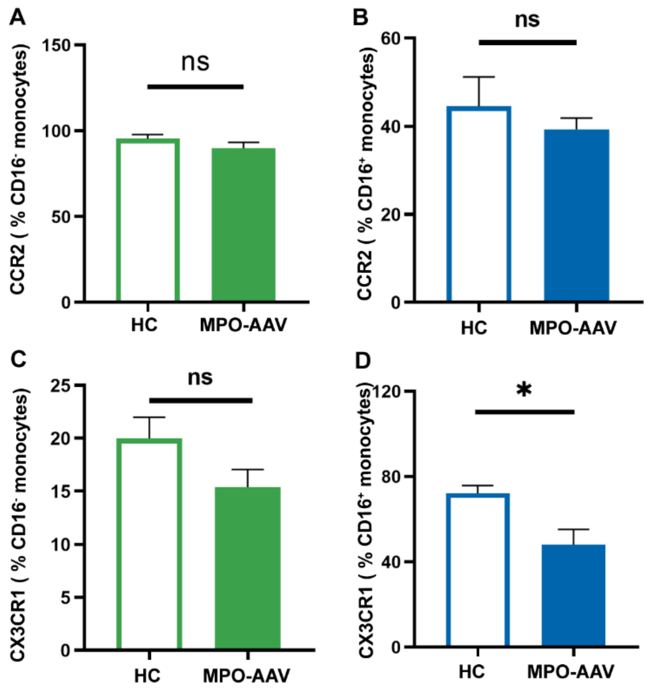

Supplementary Figure S1: The percentage of CCR2 and CX3CR1 expression on monocyte subsets of MPO-AAV patients with renal damage and HC. A) The percentage of CCR2 expression on $CD16^-$ and; B) $CD16^+$ monocytes of MPO-AAV patients and HC. C) The percentage of CX3CR1 expression on $CD16^-$ and; D) $CD16^+$ monocytes of MPO-AAV patients and HC. ns, not significant. $^*p<0.05$.

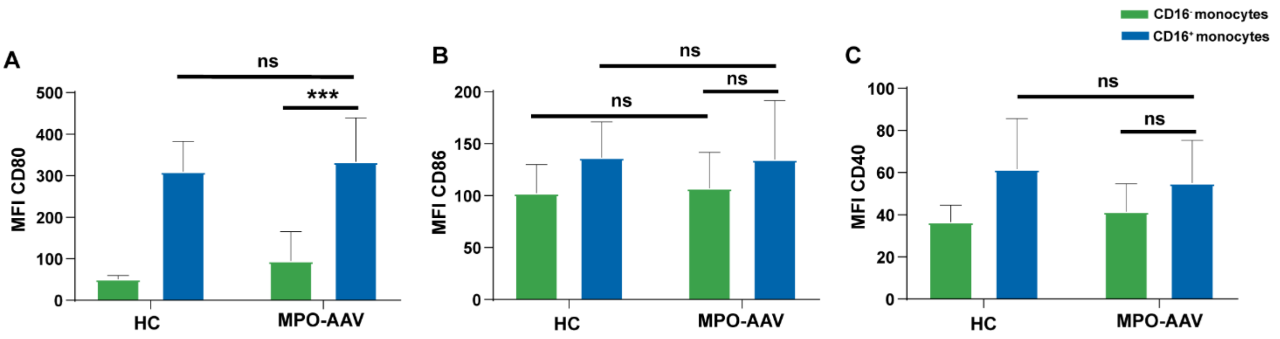

Supplementary Figure S2: Expression of CD80, CD86 and CD40 in monocyte subsets of MPO-AAV patients with renal damage and HC. A) Histogram diagrams show the mean fluorescence intensity (MFI) of CD80; B) CD86 and C) CD40 in $CD16^{+}$ and $CD16^{-}$ monocytes of MPO-AAV patients and HC. ns, not significant. ***p<0.001.

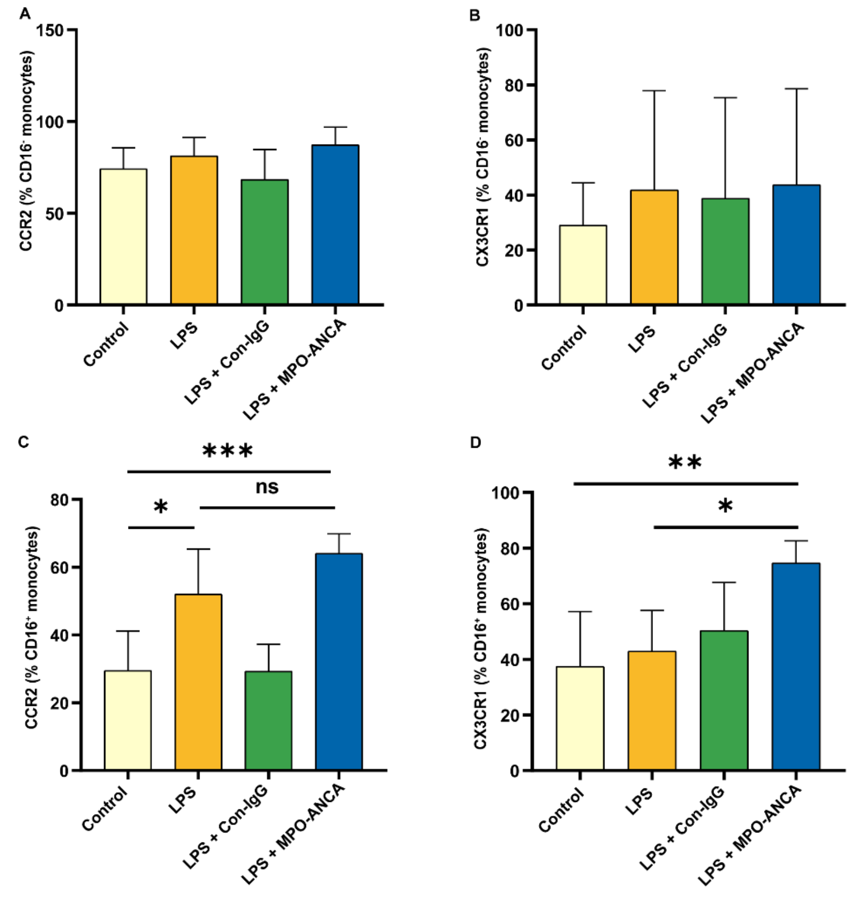

Supplementary Figure S3: The percentage of CCR2 and CX3CR1 expression on monocyte subsets in response to MPO-ANCA in vitro. A) The percentage of CCR2 and; B) CX3CR1expression on $CD16^{-}$ monocytes after stimulation. C) The percentage of CCR2 and; D) CX3CR1 expression on $CD16^{+}$ monocytes after stimulation. ns, not significant. *p<0.05; **p<0.01; ***p<0.001.

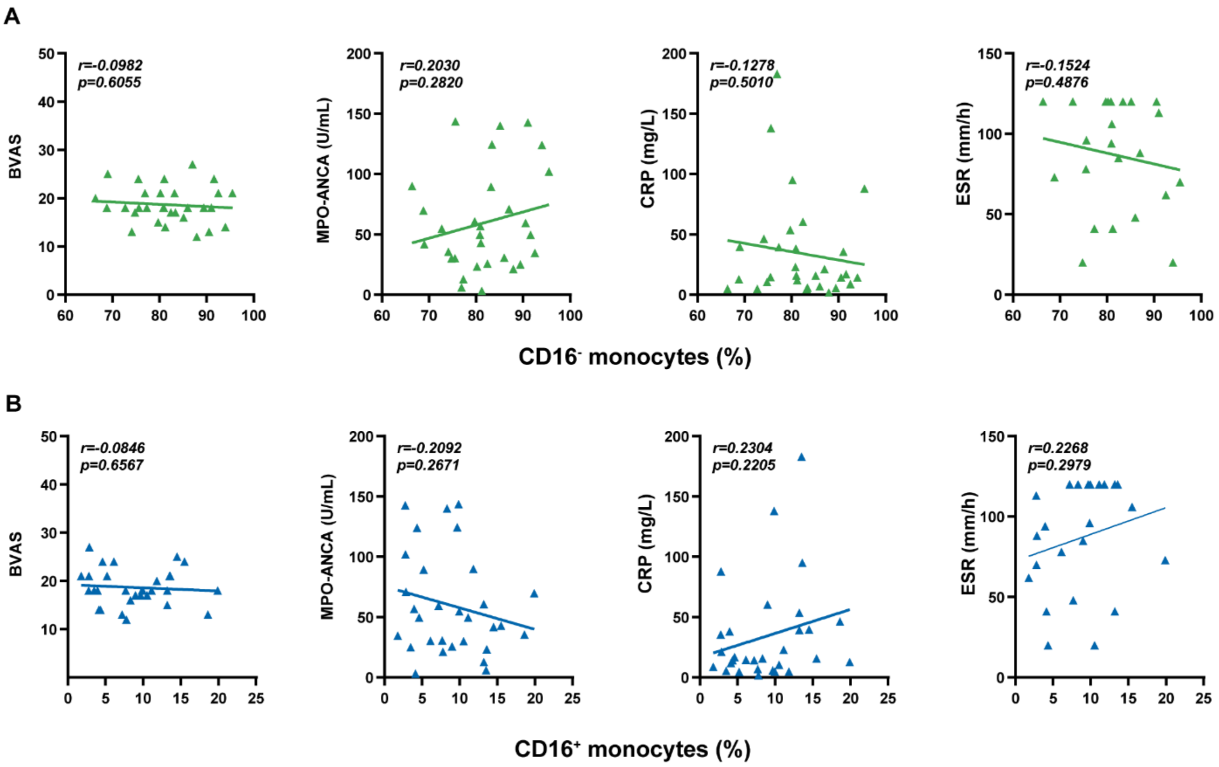

Supplementary Figure S4: Correlation between CD16^{-} and CD16^{+} monocytes abundance in blood and disease activity in MPO-AAV patients with renal damage. A) Correlations between the percentage of CD16^{-} and; B) CD16^{+} monocytes in blood and disease activity (BVAS, MPO-ANCA titers (U/mL), C-reactive protein (CRP), and erythrocyte sedimentation rate (ESR)， respectively).

1.2 Supplementary Tables

Supplementary Table S1: Clinical characteristics of patients and control

Characteristic	MPO-AAV Patients	HC	*p value*
N	143	176	
Age (years, mean ± SD)	60±14	53±11	*<0.0001*
Gender (female/male)	69/74	90/86	
Neutrophils (10^9/L)	6.81±3.36	3.49±0.83	*<0.0001*
Lymphocytes (10^9/L)	1.10±0.54	2.01±0.45	*<0.0001*
Monocytes (10^9/L)	0.61±0.27	0.39±0.09	*<0.0001*
Platelets (10^6/L)	251.73±97.86	202.88±66.61	*<0.0001*
MPO-ANCA titer (U/mL) [M±Q]	90.35 [76.06]		

Data are expressed as the number or mean ± SD. M±Q, Median±p75-p25; MPO-ANCA, myeloperoxidase- antineutrophil cytoplasmic antibody; MPO-AAV, MPO-ANCA vasculitis; HC, healthy control.

Supplementary Table S2: PCR primers

Gene	Primer
GAPDH	5' -ACAACTTTGGTATCGTGGAAGG- 3'
	5' - GCCATCACGCCACAGTTTC- 3'
CCL2	5' - CAGCCAGATGCAATCAATGCC- 3'
	5' -TGGAATCCTGAACCCACTTCT- 3'
CX3CL1	5' - GCCACAGGCGAAAGCAGTA- 3'
	5' - GGAGGCACTCGGAAAAGCTC- 3'
